# Knowledge, perceptions, and clinical experiences on molar incisor hypomineralization among dental care providers in Hong Kong

**DOI:** 10.1186/s12903-018-0678-0

**Published:** 2018-12-13

**Authors:** Gianina Camille Sicangco Gamboa, Gillian Hiu Man Lee, Manikandan Ekambaram, Cynthia Kar Yung Yiu

**Affiliations:** 1 Paediatric Dentistry, Faculty of Dentistry, The University of Hong Kong, Prince Philip Dental Hospital, 34 Hospital Road, Sai Ying Pun, Hong Kong, SAR China; 2 Paediatric Dentistry, Faculty of Dentistry, University of Otago, Dunedin, New Zealand

**Keywords:** Molar incisor hypomineralization, Disturbances in dental development, Oral health care, Dental practitioners, Children

## Abstract

**Background:**

Molar incisor hypomineralization (MIH) is an alarming problem with considerable challenges in management. This study aimed to evaluate and compare the knowledge, perceptions, and clinical experiences of molar incisor hypomineralization (MIH) between general dental practitioners (GDPs) and paediatric dentists (PDs) in Hong Kong.

**Methods:**

A cross-sectional survey of 557 randomly selected GDPs (approximately 25% of all registered dentists) and all registered PDs (*n* = 31) were invited. They were asked to complete a 4-section questionnaire adapted and modified from a study by Gambetta-Tessini and co-workers on sociodemographic profiles, knowledge, experience, and perceptions regarding MIH. Data were analyzed with chi-square, Fisher’s exact, and multiple factor ANCOVA tests.

**Results:**

The overall response rate was 43.37% (255/588). Majority (74.1%) of the respondents encountered MIH in their practices. A significantly higher mean knowledge score (46.33 ± 7.1) was observed among PDs than GDPs (43.09 ± 7.0) (*P* < 0.01) and among PDs who are 40 years old or younger (*P* < 0.001). Differences in treatment of MIH were also observed between PDs and GDPs (*P* < 0.05). Paediatric dentists were more confident in diagnosing and treating MIH (*P* < 0.001). Most respondents (87.8%) expressed a need for continuing education on MIH.

**Conclusion:**

Continuing education on MIH is needed to assure that the highest quality of evidence-based care is given to patients with MIH. Dissemination of latest best evidence on MIH, especially to GDPs, will assure that the condition is accurately diagnosed and well managed.

**Electronic supplementary material:**

The online version of this article (10.1186/s12903-018-0678-0) contains supplementary material, which is available to authorized users.

## Background

Molar incisor hypomineralization (MIH) describes the clinical appearance of demarcated enamel hypomineralization of systemic origin affecting one or more permanent first molars and occasionally the incisors [1]. Affected teeth often present an asymmetrical appearance, with one molar being severely affected while the contralateral tooth appears unaffected [2]. These defects have also been seen in the second primary molars with a similar clinical presentation [3, 4]. At present, no definitive etiological factor(s) have been identified for the occurrence of MIH. However, environmental factors including acute and chronic illnesses during the third trimester of pregnancy up to the first three years of childhood have been proposed as contributory factors to the presence of MIH [5–7].

The clinical presentation of MIH can vary from white creamy, yellow-brown demarcated enamel opacities, to post-eruptive breakdown, resembling hypoplasia or atypical caries on the enamel of the affected teeth [2]. The enamel hypomineralization seen in MIH-affected teeth occurs as a result of a disturbance during initial enamel calcification or maturation [8]. The affected enamel possesses inferior mechanical and physical properties when compared to non-affected teeth [9]. Thus, hypomineralized permanent first molars are more susceptible to post-eruptive breakdown secondary to masticatory forces acting on the teeth coupled with the dietary challenges that are present in the oral cavity [10]. This causes enamel chipping and dentine exposure as well as early dental caries. Subsequently, the overall oral health of the individual [2] will be affected.

Restoration of MIH-affected teeth is necessary not only to address the aesthetic needs of the patient, but also to address the functional and psychological needs of the child [2, 11, 12]. Management of MIH-affected teeth depends on the severity of the defect and the child’s ability to cooperate on the dental chair [13, 14]. This becomes clinically challenging due to sensitivity of the affected teeth, rapid development of caries, difficulty with anaesthesia, and repeated marginal breakdown [15]. At present, there is no evidence-based management protocol or clinical guidelines for management of MIH.

It has been difficult to determine if the condition actually presents any concern for dental care providers who are at the forefront of managing the condition. To address this need, several questionnaire-based studies on the awareness, knowledge, and perceptions of dental health care providers have been conducted in Europe [16], Australia [17, 18], New Zealand [17], Malaysia [19], and Chile [18]. Results from these studies showed that there was confusion about the prevalence, aetiology, and treatment options for MIH [16–19]. In general, hypomineralized teeth are recognized as an alarming problem with considerable challenges in terms of clinical management. There is a demand for further clinical training in the management of the condition [4, 16, 17, 20]. However, at present there is inadequate information regarding this in Hong Kong.

In Hong Kong, paediatric dentists (PDs) are trained to attend to the oral health needs of children and adolescents. Unfortunately, the number of available specialists is insufficient to meet the growing demands of the younger population in private sector. Most PDs are in the School Dental Care Service [21], which only caters to school-aged children from six to twelve years old [21]. With this scenario, the general dental practitioners (GDPs) remain as the primary dental care providers for most children in Hong Kong.

With a 2.8% reported prevalence of MIH in Hong Kong Chinese children [22], it is important to establish how often the problem is encountered, correctly identified, and managed in the clinical setting by the PDs and GDPs in Hong Kong. This research on a global problem can help local authorities strengthen their dental workforce by providing training for the dental health care providers and tailoring appropriate health services for Hong Kong Chinese children. Hence, the objective of this study was to evaluate and compare the knowledge, perceptions, and clinical experiences of MIH between the GDPs and the PDs in Hong Kong. The results of the study would be able to indicate whether MIH is considered as a clinical problem and the potential challenges encountered when managing patients affected by such condition. This study will also point out any differences in the management of MIH between the GDPs and the PDs.

## Methods

### Study sample

Two groups of dental practitioners (general dental practitioners (GDPs) and paediatric dentists (PDs)) practising in Hong Kong were recruited for this cross-sectional survey. GDPs and PDs appearing in the registry of the Dental Council of Hong Kong (DCHK) retrieved on the 11th of November 2016 were included in the study population. The register provided names and practice addresses of all registered dentists in Hong Kong. The public can access the information on the register. The dentists were consented to have their information published by DCHK. A simple random sample of 557 registered GDPs was drawn from the general register of the Dental Council of Hong Kong using a random number table. This accounted for approximately 25% of all registered Hong Kong dentists. All 33 registered PDs appearing in the list of the specialist register of the Dental Council of Hong Kong were included as well, except two who were part of the research team.

### Ethics approval and consent to participate

Ethics approval was sought from the Institutional Review Board of the University of Hong Kong/ Hospital Authority Hong Kong West Cluster (IRB Reference number: UW 160-1002) and the research was conducted in full accordance with the World Medical Association Declaration of Helsinki. Participants were consented to participate by returning the completed questionnaire. They were also consented to publish the collected data.

### Questionnaire

A questionnaire modified from Gambetta-Tessini et al. [18] was utilized for this study (Additional file 1). It included questions regarding sociodemographic data, knowledge, perceptions, clinical experience, and continuing education. Some modifications were made to fit the local context.

The questionnaire used for this study was divided into 4 sections (Additional file 1). The first section included questions on the respondents’ sociodemographic data such as gender, age, years in practice, type of practice, training, qualifications, and field of specialty if any. Specific modifications were included for the additional qualifications and training obtained by the GDPs as well as their type of practice. The second section included images of teeth affected by MIH as reference for the respondents. The third section measured the knowledge score (KS) of each respondent regarding MIH. This section included questions adapted from the original questionnaire on the participant’s awareness of MIH, prevalence, aetiology, time of insult, and the caries pattern seen in MIH. Scoring for the prevalence item was adjusted to fit the local context where the prevalence of MIH is at 2.8% [22].

The last section of the questionnaire looked into the participants’ perceptions, clinical experience, and continuing education aspects of MIH. It included questions on the type of defects most commonly encountered by the clinicians, the teeth most commonly affected, and the incidence of MIH. It also evaluated the need to refer patients for the management of MIH. Other aspects that were evaluated in this section included MIH as a dental public health issue, restorative treatments being performed, and the confidence in managing and diagnosing MIH. Additionally, an item on the preventive treatments for dental caries was added to this questionnaire. The binary nature of the question on diagnosing and managing MIH was also changed to include more options for the respondents. Items on interest in continuing education and further training regarding diagnosis, aetiology, and treatment were also added.

The questionnaire was then pilot tested on 20 taught postgraduate students under training in oral and maxillofacial surgery, endodontics, prosthodontics, and paediatric dentistry in the University of Hong Kong. Of these 20 students, 4 were registered dentists in Hong Kong and were thus excluded from the sample population of the study.

After pilot testing the questionnaire to be used for the cross-sectional survey, revisions were made to the original document. The images depicting MIH were modified as some participants thought the original images were unclear. Neutral choices were added for the questions on the confidence in diagnosing and treating MIH. Additionally, scores were adjusted for the prevalence question to appropriately reflect the prevalence of MIH in Hong Kong. No ambiguities in other questions were reported during the pilot study.

### Data collection

Each dental practitioner was sent a questionnaire via mail and a postage-paid self-addressed return envelope was provided. They also received an information sheet explaining the purpose of the study. If the practitioner voluntarily consented to participate, the completed questionnaire was returned in a sealed envelope anonymously and confidentially. A total of three mail outs, two to three weeks apart, were made. After each mail out, telephone reminders were given to the participants to maximize the response rate.

### Data analysis

All data were treated in strict confidence. Return envelopes from each participant were assigned a numerical code with the names and addresses concealed from the encoder. Data were entered and analyzed using IBM SPSS Statistics 22.0 (SPSS Inc., Chicago, IL, USA). The data obtained from the questionnaire were converted and assigned numerical coding values whenever appropriate. Simple frequency distribution of the dentists’ sociodemographic profiles and the responses to each question were tabulated. The two groups of respondents (PDs and GDPs) were compared based on their socio-demographic backgrounds, specialties, and practice profiles. Distributions and frequency tables were presented for the descriptive statistical analysis. Distributions were then compared using chi-square tests. The level of statistical significance was set at *P* < 0.05. A KS was computed based on the answers in the knowledge section of the questionnaire. The scoring method was adapted from the original questionnaire. Each answer scored a total of 9 points (Table 2). Summation of all ten answer scores resulted in a single continuous variable assigned as the KS for the participant (ranged from 20 to 60). Multiple ANOVA was used to assess the relationship between independent variables (i.e. sociodemographic background of the practitioners) and the KS. Independent variables were included in the final model when they met a threshold of *P* < 0.05 in the multivariate analyses.

## Results

A response rate of 40.93% (228/557) was achieved for the GDPs and 87.10% (27/31) for the PDs. The overall response rate of the study is 43.37% (255/588). Incomplete questionnaires with missing answers were included in the study. However, inadequately answered questions were considered as missing data.

Table 1 presents the sociodemographic profile of the respondents. There was a statistically significant difference in the age distribution between the two groups (*P* = 0.001). Most PDs (*n* = 15, 55.6%) belonged to the above 50 age group; while the GDPs were more evenly distributed across the age groups. Similarly, there was a statistically significant difference in the years of practice between the two groups (*P* = 0.001). It appears that more than half of the PDs (*n* = 15, 55.6%) have been practising longer (21-30 years) when compared to GDPs (*n* = 48, 21.1%). There was also a statistically significant difference in the types of practice between the two groups (*P* = 0.001). Most GDPs (*n* = 92, 40.9%) worked in a group private practice; while most PDs (*n* = 13, 48.1%) worked in the government. No statistically significant difference in gender was found among the two groups.

**Table 1:** Sociodemographic characteristics of GDPs and PDs in Hong Kong

Characteristics	All (*n* = 255)	GDPs (*n* = 228)	PDs (*n* = 27)	*p*-value
	n (%)	n (%)	n (%)	
Gender				
Male	163 (64.4)	146(64.6)	17 (63.0)	0.866
Female	90 (35.6)	80 (35.4)	10 (37.0)	
*Missing data*	2 (0.8)	2 (0.9)	–	
Age				
≤ 30	51 (20.2)	51 (22.6)	0 (0.0)	0.001*
31-40	63 (24.9)	61 (27.0)	2 (7.4)	
41-50	56 (22.1)	46 (20.4)	10 (37.0)	
≥ 51	83 (32.8)	68 (30.1)	15 (55.6)	
*Missing data*	2 (0.8)	2 (0.9)	–	
Years in Practice				
< 5	49 (19.3)	49 (21.6)	0 (0.0)	0.000*
6-10	33 (13.0)	33 (14.5)	0 (0.0	
11-20	61 (24.0)	56 (24.7)	5 (18.5)	
21-30	63 (24.8)	48 (21.1)	15 (55.6)	
≥ 31	48 (18.9)	41 (18.1)	7 (25.9)	
*Missing data*	1 (0.4)	1 (0.4)	–	
Type of Practice				
Solo private practice	91 (36.1)	86 (38.2)	5 (18.5)	0.000^#^
Group private practice	98 (38.9)	92 (40.9)	6 (22.2)	
Non-government organization	21 (8.3)	19 (8.4)	2 (7.4)	
Government	34 (13.5)	21 (9.3)	13 (48.1)	
Hospital	8 (3.2)	7 (3.1)	1 (3.7)	
*Missing data*	3 (1.2)	3 (1.3)	–	

*MIH* molar incisor hypomineralization, *GDPs* general dental practitioners, *PDs* paediatric dentists

*Statistically significant (*P* < 0.05), Pearson’s chi-square (χ^2^) test

^#^Statistically significant (*P* < 0.05) Fisher’s exact test

Percentage distributions for the questions included in section 3 of the questionnaire are listed in Table 2. The mean KS for all the respondents was 43.44 ± 7.0, with a range of 21-59. The difference in the mean KS for GDPs (43.09 ± 7.0, range: 21-59) and PDs (46.33 ± 7.1, range: 33-59) was statistically significant at *P* < 0.05. More PDs (*n* = 26, 96.3%) were aware that MIH is a developmental defect different from fluorosis and hypoplasia when compared to GDPs (*n* = 166, 72.8%) (*P* < 0.01). Almost half of the respondents (*n* = 117, 46.1%) perceived the prevalence of MIH to be less than 5% in the HK population. Also, more GDPs (*n* = 161, 70.6%) considered genetic factors as one of the aetiologies of MIH when compared to PDs (*n* = 14, 51.9%) (*P* < 0.05). Both GDPs and PDs had a variety of views with regards to time or period when the insults leading to MIH occurred. Over half of the GDPs (*n* = 149, 65.9%) and PDs (*n* = 16, 59.2%) indicated that the insults leading to MIH could have been present since pregnancy; while one-third of the GDPs (*n* = 77, 34.1%) and PDs (*n* = 11, 40.1%) postulated that the insults leading to MIH could have appeared only after birth (in the first/third year of life). Majority of the GDPs (*n* = 145, 65.9%) and PDs (*n* = 22, 81.5%) agreed that the pattern of caries related to MIH was different from the classical caries pattern (*P* = 0.174).

**Table 2:** Percentage distribution of knowledge scores of GDPs and PDs for each question regarding MIH knowledge

Knowledge questions		Knowledge Scores		Percentage distribution of practitioners answered ‘YES’			*p*-value
		Yes	No	All n (%)	GDPs n (%)	PDs n (%)	
Have you been aware that MIH is a developmental defect that differs from fluorosis and hypoplasia?		9	0	192 (75.3)	166 (72.8)	26 (96.3)	0.007*
How prevalent do you think MIH might be in your community? (One option chosen)	< 5%	6	^	117 (46.1)	102 (44.9)	15 (55.6)	0.422
	5-10%	1	^	72 (28.3)	63 (27.8)	9 (33.3)	
	10-20%	1	^	25 (9.8)	23 (10.5)	2 (7.4)	
	> 20%	1	^	8 (3.5)	8 (3.1)	0 (0.0)	
	Not sure	0	^	32 (12.6)	31 (13.7)	1 (3.7)	
Do you think they are involved in the aetiology of MIH?	Genetic factors	5	4	175 (68.6)	161 (70.6)	14 (51.9)	0.019^#^
	Environmental contaminants	5	4	118 (46.3)	104 (45.6)	14 (51.9)	0.539
	Chronic medical conditions affecting mother and child	6	3	206 (80.8)	188 (82.5)	24 (88.9)	0.587
	Acute medical conditions affecting mother or child	6	3	206 (80.8)	182 (82.5)	24 (88.9)	0.587
	Antibiotics or medications	5	4	106 (41.6)	98 (43.0)	8 (29.6)	0.183
	Fluoride exposure	1	8	61 (23.9)	56 (24.6)	5 (18.5)	0.486
What time/period do you think the insult occurs? (one option chosen)	During pregnancy	1	^	25 (9.9)	25 (11.1)	0 (0.0)	0.451
	1st year of life	3	^	60 (23.7)	52 (23.0)	8 (29.6)	
	3rd year of life	0	^	28 (11.1)	25 (11.1)	3 (11.1)	
	Pregnancy to 1st year of life	3	^	55 (21.7)	48 (21.2)	7 (25.9)	
	Pregnancy to 3rd year of life	2	^	85 (33.6)	76 (33.6)	9 (33.3)	
Do you think the pattern of caries related to MIH is different from the classical caries pattern?		7	1	167 (65.5)	145 (65.9)	22 (81.5)	0.174
Mean Knowledge Score (SD)				43.44 (7.0)	43.09 (7.0)	46.33 (7.1)	0.024**
Ranges		Min 20	Max 60	21-59	21-59	33-59	
Total sample				255	228	27	

*MIH* molar incisor hypomineralization, *GDPs* general dental practitioners, *PDs* pediatric dentists

^Answer “No” does not apply as it was analyzed as a single choice question

*Statistically significant (*P* < 0.05), Pearson’s chi-square (χ2) test

^#^Statistically significant (*P* < 0.05) Fisher’s exact test

^**^Statistically significant difference (*P* < 0.05) independent sample t-test

Data on perceptions, clinical experience, and continuing education aspects of dental practitioners regarding MIH are presented in Table 3. Majority of the respondents had encountered MIH in their practices (*n* = 198, 77.6%). Significantly more PDs (*n* = 26, 96.3%) had encountered MIH compared to GDPs (*n* = 172, 75.4%) (*P* < 0.05). Practitioners reported that ‘yellow/brown demarcations’ was the most frequent type of defect they had encountered in their practices (GDPs: *n* = 104, 46.2%, PDs: *n* = 10, 37.0%). A similar percentage of practitioners reported that they had seen white demarcated defects (GDPs: *n* = 52, 23.1%, PDs: *n* = 8, 29.6%) and post-eruptive breakdown (GDPs: *n* = 48, 21.3%, PDs: *n* = 9, 33.3%) in their practices. More than half of the PDs (*n* = 18, 66.7%) have observed hypomineralized lesions on premolars compared to fewer than half of the GDPs (*n* = 92, 40.5%) (*P* < 0.05). More PDs (*n* = 10, 38.5%) noticed same defects in primary dentition compared to GDPs (*n* = 49, 21.6%) (*P* > 0.05). Also, most GDPs (*n* = 132, 57.9%) considered referring a child who has signs of MIH to a paediatric dental specialist. Similarly, most specialists (*n* = 14, 51.9%) felt that MIH is best managed by a paediatric dental specialist. Over two-fifths of GDPs (*n* = 98, 43.2%) and half of PDs (*n* = 15, 55.6%) agreed that MIH would be the next public health problem after dental caries.

**Table 3:** Perceptions, clinical experience, and continuing education aspects of GDPs and PDs regarding MIH

Questions		All n (%)	GDPs n (%)	PDs n (%)	*p*-value
Do you encounter teeth with MIH in your practice? (YES)		198 (77.6)	172 (75.4)	26 (96.3)	0.025^#^
What is/are the most frequent type of defect seen in your practice?	White demarcated	60 (23.8)	52 (23.1)	8 (29.6)	0.177
	Yellow/brown demarcations	114 (45.2)	104 (46.2)	10 (37.0)	
	Post-eruptive breakdown	57 (22.6)	48 (21.3)	9 (33.3)	
	None	21 (8.3)	21 (9.3)	0 (0.0)	
In what other permanent teeth have you encountered MIH-like defects?	Premolars	110 (43.3)	92 (40.5)	18 (66.7)	0.010*
	Second permanent molars	63 (24.7)	55 (24.1)	8 (29.6)	0.530
	Canines	67 (26.3)	61 (26.8)	6 (22.2)	0.613
Do you notice these defects in the primary dentition? (YES)		59 (23.3)	49 (21.6)	10 (38.5)	0.054
Do you feel the incidence has increased in the period of your practice? (YES)		40 (15.7)	33 (14.5)	7 (25.9)	0.157
Would you refer a child who has signs of MIH to a paediatric dental specialist?	Yes or when possible	146 (57.3)	132 (57.9)	14 (51.9)	0.548
	No	109 (42.7)	96 (42.1)	13 (48.1)	
Do you think MIH represents a clinical problem that could come next to dental caries in public health? (YES)		113 (44.5)	98 (43.2)	15 (55.6)	0.221
What type of preventive treatment do you often use to treat these teeth? (Only those YES answers are shown)	Fluoride varnish	215 (84.6)	189 (83.3)	26 (96.3)	0.091
	Silver diamine fluoride solution	24 (9.4)	19 (8.3)	5 (18.5)	0.157
	Tooth mousse	90 (35.3)	79 (34.6)	11 (40.7)	0.531
	Fissure sealant	107 (42.0)	90 (37.5)	17 (63.0)	0.019*
What type of treatment do you often use to treat MIH?	Microabrasion	29 (11.4)	27 (11.8)	2 (7.4)	0.493
	Resin infiltration	46 (18.0)	44 (19.3)	2 (7.4)	0.185
	Glass ionomer	123 (48.2)	108 (47.4)	15 (55.6)	0.421
	Composite	171 (67.1)	150 (65.8)	21 (77.8)	0.210
	Amalgam	14 (5.5)	11 (4.8)	3 (11.1)	0.174
	Preformed crowns	62 (24.3)	51 (22.4)	11 (40.7)	0.035*
	Extraction	19 (7.5)	12 (5.3)	7 (25.9)	0.001^#^
How do you feel about diagnosing MIH?	Very confident	20 (7.8)	11 (4.8)	9 (33.3)	0.000^#^
	Confident	132 (51.8)	116 (50.9)	16 (59.3)	
	Unconfident	91 (35.7)	89 (39.0)	2 (7.4)	
	Very unconfident	12 (4.7)	12 (5.3)	0 (0.0)	
How do you feel about treating MIH?	Very confident	10 (4.0)	7 (3.1)	3 (3.7)	0.000^#^
	Confident	109 (43.1)	89 (39.4)	20 (74.1)	
	Unconfident	123 (44.6)	119 (52.7)	4 (14.8)	
	Very unconfident	11 (4.3)	11 (4.9)	0 (0.0)	
Are you receiving any information on MIH? (YES)		35 (13.7)	20 (8.8)	15 (55.6)	0.000^#^
Would you like further training regarding tooth hypomineralization?	Diagnosis	150 (58.8)	141 (64.9)	9 (33.3)	0.002*
	Aetiology	147 (57.6)	134 (58.8)	13 (48.1)	0.298
	Treatment	215 (84.3)	192 (84.2)	23 (85.2)	1.000
	No training	31 (12.2)	27 (11.8)	4 (14.8)	0.757

Results may not add due to missing values

*GDPs* general dental practitioners, *PDs* pediatric dentists, *MIH* molar incisor hypomineralization

*Statistically significant (*P* < 0.05), Pearson’s chi-square (χ2) test

# Statistically significant (*P* < 0.05) Fisher’s exact test

Table 3 also shows the percentage distributions for preventive and restorative treatment strategies for GDPs and PDs. Significantly more PDs (*n* = 17, 63.0%) consider placing fissure sealants as part of their preventive treatment when compared to GDPs (*n* = 90, 37.5%) (*P* < 0.05). Similarly, significantly more PDs opt to restore with preformed crowns (*P* < 0.05) or perform extraction (*P* = 0.001) for teeth affected with MIH when compared to GDPs. Only about 5% (*n* = 12) of GDPs would consider extraction as a treatment option for the management of MIH-affected teeth.

In terms of the confidence in diagnosing MIH, over 90% of the PDs (*n* = 25, 92.6%) were confident or very confident in diagnosing MIH; while only a little over half of the GDPs (*n* = 127, 55.7%) were confident enough to diagnose MIH (*P* < 0.001) (Table 3). Likewise, most PDs (*n* = 23, 77.8%) were confident or very confident in treating MIH; while only over two-fifths of the GDPs (*n* = 96, 42.5%) were confident (*P* < 0.001). A good number of PDs (*n* = 15, 55.6%) reported receiving some information on MIH; while only 8.8% (*n* = 20) of the GDPs reported receiving some information on MIH (*P* < 0.001). Most of the respondents would like to have further training on MIH (Table 3), in which significantly more GDPs (*n* = 141, 64.9%) wanted to have the training specifically on diagnosis of MIH compared to PDs (*n* = 9, 33.3%) (*P* = 0.002). Over 80% of GDPs (*n* = 192, 84.2%) and PDs (*n* = 23, 85.2%) indicated that they would like to have further training in treating MIH affected teeth (*P* > 0.05).

The final multiple regression model with key factor found KS to be influenced by the practitioners’ sociodemographic profile as presented in Table 4. The oral health care practitioners group (general dental practitioners or paediatric dentists) (B coefficient = − 4.608, *P =* 0.002, with PDs as the reference group) and the age group of practitioners (≥41 years old) (B coefficient = 3.212, *P* < 0.001) remained as the predictors of the knowledge score. PDs had significantly higher KS when compared to GDPs (*P* = 0.002). Likewise, PDs and GDPs from the younger age group (≤40 years old) reported significantly higher KS (*P* < 0.001).

**Table 4:** Final Multiple ANOVA model for the knowledge score among oral health care practitioners

Independent Variables	B	Std. Error	*p*-value	95% Confidence interval	
				Lower boundary	Upper boundary
Constant	46.095	1.322	0.000	43.492	48.698
Group					
GDPs	−4.608	1.447	0.002^*^	−7.457	−1.758
PDs^					
Age					
≤ 40 (1)	3.212	0.898	0.000	1.444	4.980
≥ 41(2)^					

*GDPs* general dental practitioners, *PDs* paediatric dentists, *MIH* molar incisor hypomineralization

*Independent variables with *P* < 0.05 after multivariable analysis with multiple ANOVA were included in the final model

^Reference group

## Discussion

This is the first study conducted to explore the knowledge, perceptions, and clinical experiences on MIH among the dental care providers in Hong Kong. This study is essential in establishing the level of knowledge of both GDPs and PDs in the area. It highlights any knowledge gaps that need to be addressed to improve the delivery of care in terms of diagnosis and management of MIH in Hong Kong population.

In this study, the total number of participants recruited was 588. A sample of 557 GDPs accounting for 25% of the total GDP population (the usual sampling percentage for randomised surveys) and all 31 PDs who were not part of the research team (for the total number of PDs in Hong Kong is small) were included in the study. An overall response rate of 43.37% (255/588) was achieved in this study. Multiple reminders (a total of 3 mailing rounds and at least 3 phone call reminders in between the mailings) helped in maximizing the response rate.

The sample in this study included a representative sample of both GDPs and PDs. The sociodemographic profile of the respondents in this study is comparable to that of the dentist population of Hong Kong [23]. Hence, the recruited sample would provide a valid assessment of the knowledge, perceptions, and clinical experiences on MIH among the dental care practitioners in Hong Kong.

When compared to the response rate of the PDs, a relatively lower response rate of 40.93% for the GDPs was obtained in the present study. Similar cross-sectional surveys on MIH had a response rate of 58.2% for GDPs and dental nurses in Malaysia [19], 58.8% for GDPs, PDs, students, and dental therapists in Australia and New Zealand [17], and 29% for GPDs and oral health therapists in Chile and Australia [18].

Results from this cross-sectional survey showed that most of the dentists in Hong Kong have encountered MIH in their practices. These findings are consistent with those reported in the previous studies [4, 16, 17]. The general prevalence of MIH in HK has been low (2.8%) based on a single study done by Cho et al. [22]. However, the true prevalence of MIH may have been underestimated due to the inherent limitations of a retrospective study. The condition may have been underreported in the dental records which were reviewed. The differences in the age and ethnicity of the subjects may have likewise contributed to the lower prevalence rate in Hong Kong. It was reported that children with hypomineralized second primary molars or primary canines would have a higher chance of getting MIH. There is no study reporting the increased odds in children in Hong Kong.

Significantly more PDs have reported encountering teeth with MIH which is consistent with that reported by Gambetta-Tessini et al. [17]. This could be due to the higher number of paediatric patients being seen in their practices when compared to GDP practices which have a smaller proportion of paediatric patients in their patient pool. Since MIH lesions are more commonly seen as atypical caries later on in life, GDPs may have misdiagnosed them as dental caries rather than post-eruptive breakdown secondary to enamel hypomineralization. Yellow-brown demarcations were the most frequently observed lesion by the respondents. This could be due to the unique and obvious presentation of the lesion which cannot be confused with dental caries, white spot lesions, or enamel hypoplasia [4].

A significant difference in the KS between GDPs and PDs was also reported in this study (*P* < 0.01). Results from the study show that PDs had higher KS compared to GDPs. Likewise, more PDs (96.3%) were aware that MIH differs from fluorosis and enamel hypoplasia (*P* < 0.05). This could be explained by the findings in Table 3 where more than half of the PDs (55.6%) have reported receiving some information regarding MIH compared to only 8.8% of GDPs having received information on MIH. The relatively low percentage of information on MIH for GDPs is seen in other countries like Malaysia, where only 7% of GDPs received information on MIH [19].

Clinical decision making is a multifactorial process which can be affected by the dentist, patient, and health care system issues [24]. Based on the multiple factor regression model, both age and specialty qualification affect the KS. PDs were shown to have higher KS when compared to GDPs. Younger practitioners who were aged 40 years old or below also reported higher KS. Since the diagnostic criteria for MIH were only introduced in 2001 [1], the older generation of dentists may not have had access to any information regarding MIH in the dental school. Since available information regarding MIH is also very scarce (with only less than 15% for the sample population), they may also have limited access to the knowledge even after graduation.

Recently published studies have reported the presence of similar defects in the primary dentition and their presence has been associated with an increased risk of developing MIH [3, 4]. However, only 23.3% of the respondents have observed the defect in primary teeth. A higher percentage of PDs have reported observing MIH-like lesions in the primary dentition although the difference is not significant. This could be attributed to the higher frequency of paediatric patients that are seen in their practices, higher knowledge or awareness of the problem, as well as better access to information on MIH among the PDs. With that, stronger information campaigns on MIH should be provided to all dental practitioners so that the dentist can identify these red flags early on. When dentists are better informed, they could warn parents of the increased risk of developing MIH in patients who present with similar defects in the primary dentition. The attending dentist will then be able to perform closer monitoring of the erupting first permanent molars and incisors so that properly timed interventions can be provided for the affected teeth. This allows the clinician to enforce preventive measures to minimize post-eruptive breakdown and prevent caries or arrest caries progression of the compromised teeth.

Available treatment options for the management of MIH include fluoride varnish, remineralising sugar-free dental topical cream containing casein phosphopeptide amorphous calcium phosphate (CPP-ACP), fissure sealant, microabrasion, resin infiltration, glass ionomer, composite, amalgam, preformed crowns, and extractions [10, 13]. From the results in Table 3, more PDs reported using fissure sealants (*P* < 0.05), preformed crowns (*P* < 0.05), and extractions (*P* = 0.001) to manage MIH. These findings may reflect the difference in the training received by the GDPs and PDs. Arguably more information on the management of MIH is received by the PDs during their postgraduate training courses. Therefore, more PDs also feel confident in diagnosing and managing MIH alike (*P* < 0.001).

A significantly higher percentage of dental health care professionals (87.8%) have expressed their interest for further training on the diagnosis, aetiology and treatment of MIH. As mentioned, the information being received by the PDs on MIH is already sufficient thus eliminating the need for further training on some aspects of MIH. As dental health care professionals, part of our responsibilities is to keep ourselves up-to-dated with the growing body of knowledge and new information that are available. This will allow to practice evidence-based dentistry and provide the highest quality of care for the patients.

Clinical guidelines on the management of MIH should be made available for all practising dentists in Hong Kong. It needs to be implemented and reinforced with continuing education seminars on how to use and apply the guidelines. This will greatly improve and standardize the diagnosis and management of MIH in Hong Kong. More GDPs can be made more aware of the problem, equipped to properly diagnose the condition, and treat simple cases for secondary care. In such situation, the utmost priority will be the well-being of the patients.

A limitation of the study design is that the questionnaires in this study were sent by post and responses were self-reported, therefore it was subjected to response bias. However, when using randomised surveys for the assessment of professional practice, the outcome is hardly biased by non-response [25]. Another limitation is the difficulty to involve dental practitioners in research and response rates are usually low in most places [26]. Hence, caution must be exercised when generalizing the results from the present study to the entire GDPs population in Hong Kong. The inherent limitations of a cross-sectional survey should also be taken into consideration when interpreting the results of this study.

## Conclusions

Molar incisor hypomineralization is a common oral health condition frequently encountered by both GDPs and PDs in Hong Kong and elsewhere. A higher knowledge score was observed for PDs compared to GDPs. More PDs received information on MIH and are more confident in the diagnosis and treatment of MIH. Variations in the encounter of MIH in their practices, as well as the preventive, restorative, and surgical treatment modalities available to the patients were also observed. Most dental practitioners expressed a need for further training on the diagnosis, aetiology, and treatment of MIH. Continuing education on MIH is needed to assure that the highest quality of evidence-based care is given to patients with MIH. Latest information dissemination, especially to GDPs, will assure that the condition is accurately diagnosed and managed.

## Additional file

Additional file 1: The 4-section questionnaire adapted and modified from Gambetta-Tessini et al. [18]. (DOC 7080 kb)

## Abbreviations

ANCOVA: Analysis of Covariance
ANOVA: Analysis of Variance
GDPs: General Dental Practitioners
IRB: Institutional Review Board
KS: Knowledge Score
MIH: Molar Incicsor Hypomineralization
OHCPs: Oral Health Care Practitioners
PDs: Paediatric Dentists
