# Supplementary material for: Knowledge, perceptions, and clinical experiences on molar incisor hypomineralization among dental care providers in Hong Kong
Source: BMC Oral Health. 2018 Dec 13;18:217. doi: 10.1186/s12903-018-0678-0 (PMC6293514; doi:10.1186/s12903-018-0678-0)
Supplement: Supplementary file 1 — The 4-section questionnaire adapted and modified from Gambetta-Tessini et al. [18]. (DOC 7080 kb) [file 12903_2018_678_MOESM1_ESM.doc]

Figure Legends

| Fig. 1 | The 4-section questionnaire adapted and modified from Gambetta-Tessini et al. (2016). |  |
| --- | --- | --- |

**Fig. 1. The 4-section questionnaire adapted and modified from Gambetta-Tessini et al. (2016).**

**Please tick ✔ as appropriate for answers with selection boxes provided.**

**
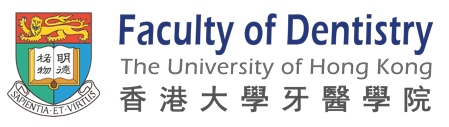
**

| **Socio-demographic background and practice profil**e |
| --- |
| 1. **Your gender**    - Male    - Female 2. **Age***    - ≤30    - 31-40    - 41-50    - ≥51 3. **How long have you been practicing dentistry?***    - Less than 5 years    - 5 to 10 years    - 11 to 20 years    - 21 to 30 years    - More than 30 years 4. **What is the type of your practice? (if you have more than one job, please indicate the type of practice in which you spent most of your working time.)***    - Solo private practice    - Group private practice    - Non-government organization    - Government    - Hospital    - Others, please specify_____________________ 5. **Was there adequate training in dental school regarding management for hypomineralized teeth?**    - Yes    - No 6. **Did you obtain any additional qualification(s), which is/are relevant to the dental profession?***    - Yes, please specify: (you may tick **✔** more than one box below.)      - Post-graduate Certificate      - Post-graduate Diploma      - Master’s degree      - Doctoral degree      - Fellowship      - Membership      - Others, please specify: _____________________________    - No 7. **Are you a dental therapist, general practitioner, specialty trainee or specialist?^#^**    - Dental therapist    - General practitioner    - Postgraduate Student under speciality training, please specify your field below.    - Trainee, please specify your field below.    - Specialist, please specify your field below.      - Community Dentistry      - Endodontics      - Family Dentistry      - Oral and Maxillofacial Surgery      - Orthodontics      - Paediatric Dentistry      - Periodontology      - Prosthodontics |

| MIH Images |
| --- |
| The following questions relate to these images.*  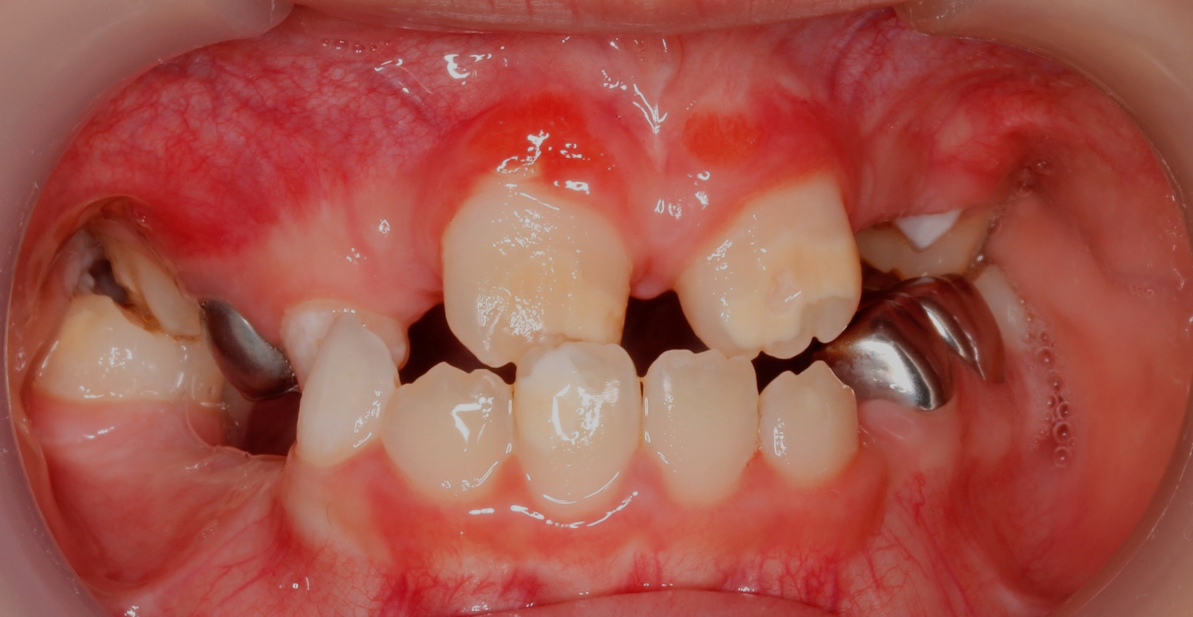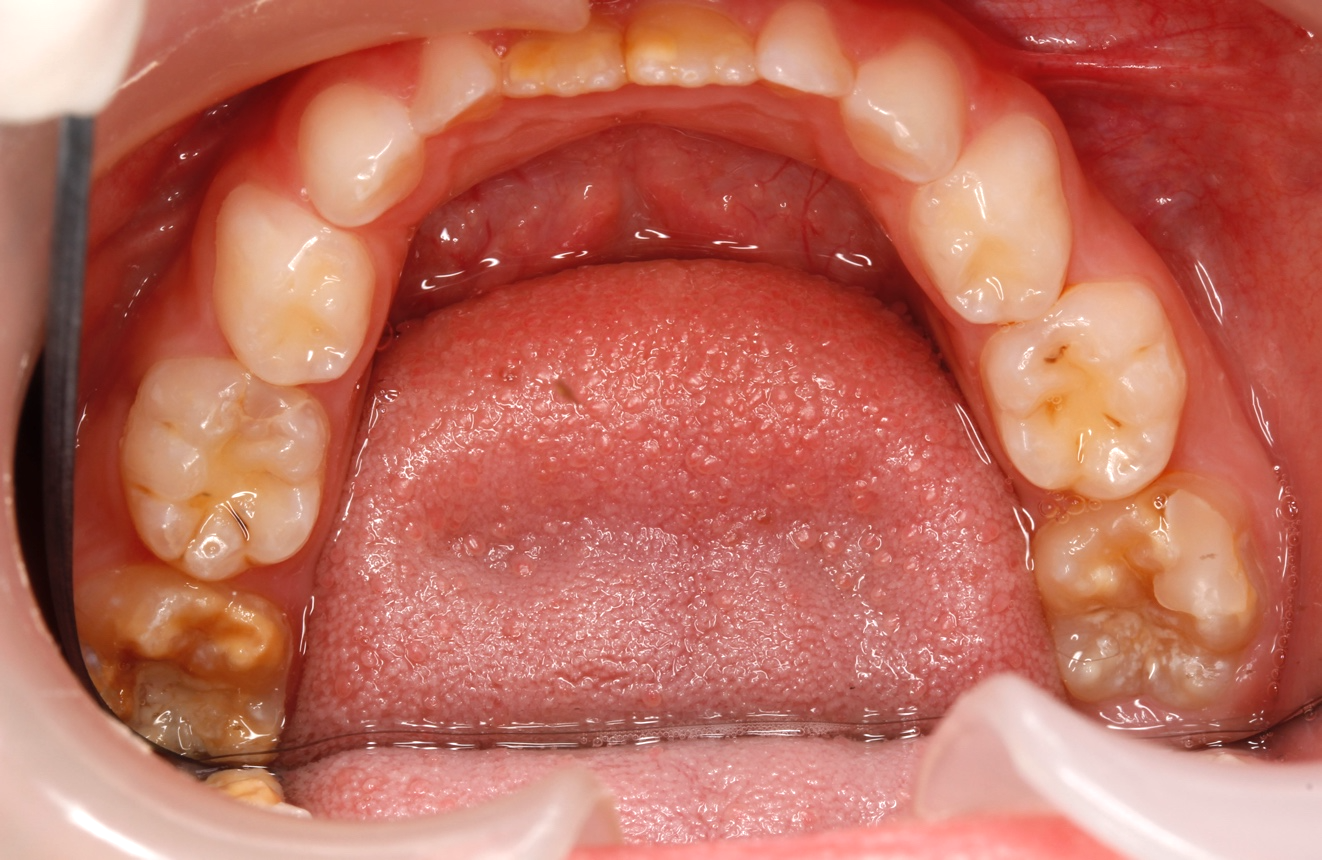**Images of Hypomineralized First Permanent Molars and Incisors**  **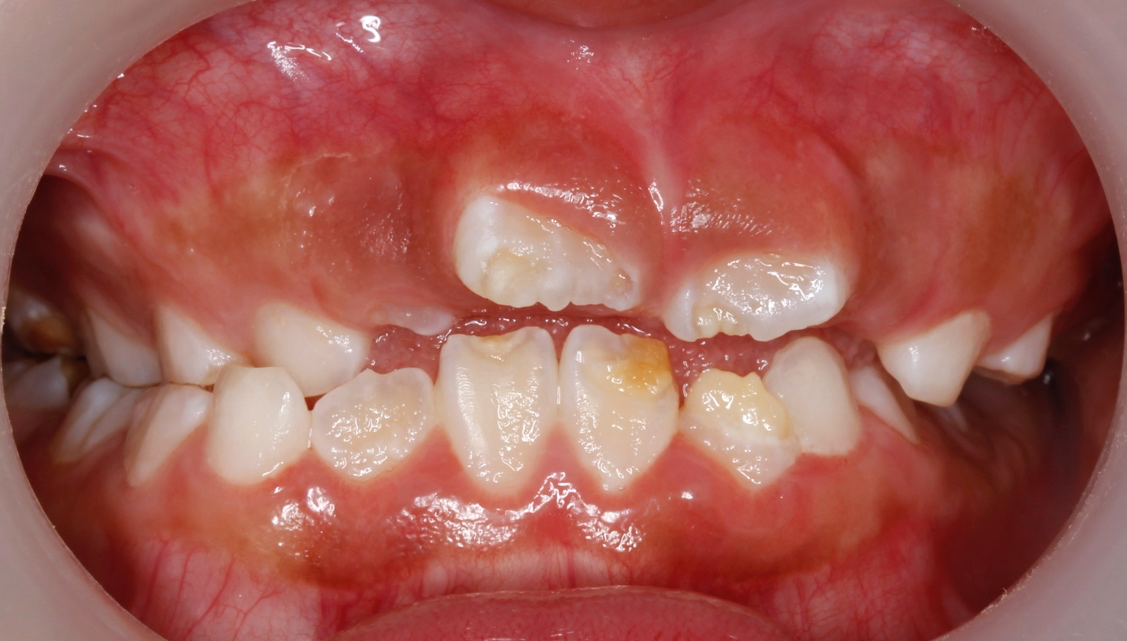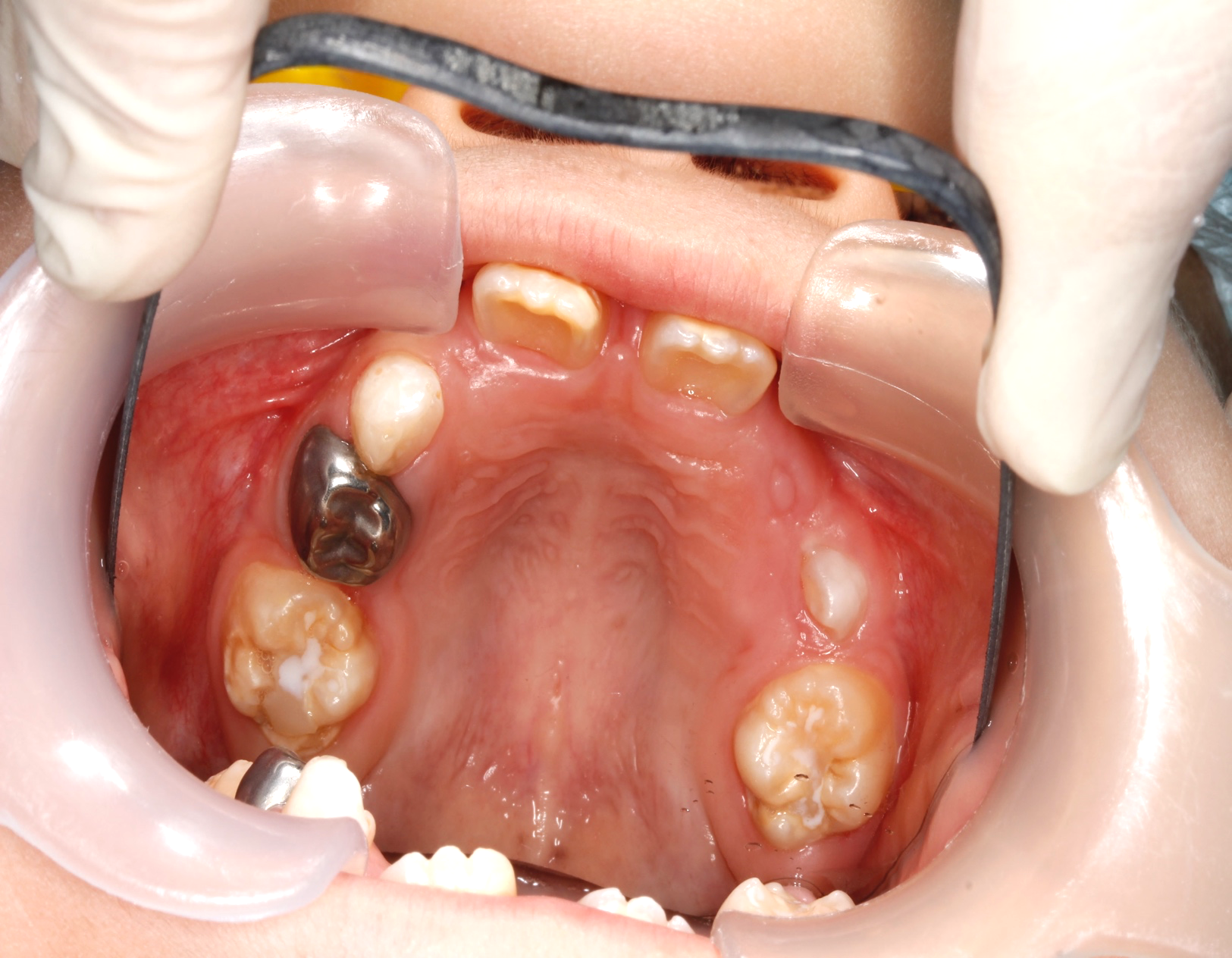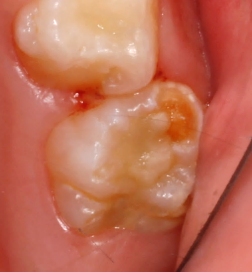** |
|  |

| **Knowledge** |
| --- |
| **1. Have you been aware that MIH is a developmental defect that differs from fluorosis and hypoplasia?**   - Yes - No   **2. How prevalent do you think MIH might be in your community? (One option chosen)**   - <5% - 5 – 10 % - 10 – 20 % - >20% - Not sure   **3. Do you think they are involved in the aetiology of MIH? (you may tick ✔ more than one box below.)**   - Genetic factors - Environmental contaminants - Acute medical conditions affecting mother or child - Chronic medical conditions affecting mother or child - Antibiotics or medications - Fluoride exposure   **4. During what time/period do you think this insult occurs?**   - During pregnancy - 1^st^ year of life - 3^rd^ year of life - Pregnancy to 1^st^ year of life - Pregnancy to 3^rd^ year of life   **5. Do you think the pattern of caries related to MIH is different from the classical caries pattern?**   - Yes - No - Not sure |

| **Perception, clinical experience, continuing education** |
| --- |
| **1. Do you encounter teeth with MIH in your practice?**   - Yes - No   **2. What is the most frequent type/ defect seen in your practice?**   - White demarcated - Yellow/ brown demarcations - Post-eruptive breakdown - None   **3. In what other permanent teeth have you encountered MIH-like defects? (you may tick ✔ more than one box below.)**   - Premolars - Second permanent molars - Canines - None   **4. Do you notice these defects in the primary dentition?**   - Yes - No   **5. Do you feel the incidence has increased in the period of your practice?**   - Yes - No   **6. Would you refer a child who has signs of MIH to a paediatric dental specialist for treatment?**   - Yes or when possible - No   **7. Do you think MIH represents a clinical problem that could come next to dental caries in public health?**   - Yes - No   **8. What type of preventive treatment do you often use to treat these teeth? (you may tick ✔ more than one box below.)^#^**   - Fluoride varnish - Silver diamine fluoride solution - Tooth mousse - Fissure sealant - Others: (please specify) ___________________   **9. What type of treatment you often use to treat MIH? (you may tick ✔ more than one box below.)***   - Microabrasion - Resin infiltration - Glass ionomer - Composite - Amalgam - Preformed crowns - Extraction   **10. How confident do you feel when diagnosing MIH teeth?^#^**   - Very confident - Confident - Unconfident - Very unconfident   **11. How confident do you feel in managing MIH teeth?^#^**   - Very confident - Confident - Doubtful - Unconfident  1. **Are you receiving any information on MIH?**    - Yes    - No 2. **Would you like further training regarding tooth hypomineralization? (you may tick ✔ more than one box below.)^#^**  - Diagnosis - Aetiology - Treatment - No training |

**Thank you for your collaboration with this project.**

*Questions modified from the original questionnaire

^#^Questions added to the original questionnaire
